# The Complex Association Between Bariatric Surgery and Depression: a National Nested-Control Study

**DOI:** 10.1007/s11695-020-05201-z

**Published:** 2021-02-03

**Authors:** Chanpreet Singh Arhi, Roise Dudley, Osama Moussa, Maddalena Ardissino, Samantha Scholtz, Sanjay Purkayastha

**Affiliations:** Department of Surgery and Cancer, Imperial College London, St Mary’s Hospital, London, W2 1NY UK

**Keywords:** Bariatric surgery, Depression, Primary care, CPRD

## Abstract

**Background:**

Although bariatric surgery has been shown to reduce weight loss and obesity-related conditions, an improvement in depression remains unclear. The aim of this study was to determine whether bariatric surgery is associated with a resolution of depression, and the prevention of its onset.

**Method:**

Patients with a BMI ≥ 30 kg/m^2^ who had undergone bariatric surgery were identified from the Clinical Practice Research Datalink (CPRD), matched 5:1 to controls. Cox regression analysis was used to determine the risk of developing de novo depression. Kaplan-Meier analysis compared the proportion of patients with no further consultations related to depression between the two groups.

**Results:**

In total, 3534 patients who underwent surgery, of which 2018 (57%) had pre-existing depression, were matched to 15,480 controls. Cox proportional hazard modelling demonstrated surgery was associated with a HR of 1.50 (95% CI 1.32–1.71, *p* < 0.005) for developing de novo depression. For those with pre-existing depression, by 5 years, just over 20% of post-surgical patients had no further depression episodes compared with 17% of controls.

**Conclusion:**

In individuals with a history of depression, bariatric surgery is associated with an improvement in mental health. On the contrary, the finding of increased de novo diagnoses of depression following surgery indicates the need for further study of the mechanisms by which bariatric surgery is associated with depression in this subset of patients.

**Supplementary Information:**

The online version contains supplementary material available at 10.1007/s11695-020-05201-z.

## Introduction

It is predicted that by 2030, 46% of men and 40% of women will be obese in the UK [1]. These figures represent a rapidly escalating public health crisis which affects both low- and high-income countries and an increasingly severe detriment to health-related quality of life worldwide [2]. The universal measure used to assess weight classifications is the body mass index (BMI) in which a value ≥ 25 kg/m^2^ confers an increased risk of obesity-associated health conditions [2]. These include, but are not limited to, cancer, cardiovascular disease, metabolic syndrome and depression [3].

Depression is a significant cause of economical disease burden worldwide [4]. Increasing severity of depression is also associated with greater all-cause mortality including cardiovascular event and stroke [5]. Although there are many causes of depression, there is a well-established link between obesity and depression synergistically decreasing both physical and mental quality of life [6] as affected individuals enter a vicious cycle; worsening depression is associated with weight gain which, in turn, leads to worse depression outcomes [7]. As a directly proportional relationship between risk of depression and obesity becomes established in the literature [8], the need for a treatment that can address both conditions is increasingly clear.

Bariatric surgery is a highly efficacious intervention for obesity, both in terms of overall weight loss and resolution of weight-associated comorbidities [9]. Furthermore, a recent review of the literature illustrates a significant reduction in depression severity and long-term psychological improvements following bariatric surgery [10].

However, while this review comments on the potential of bariatric surgery as a therapeutic tool in treating depression, it is limited by the low number of studies which use depression as a primary outcome [10]. Our retrospective nested-control study aims to evaluate the impact bariatric surgery has on depression, including the possibility to reduce the risk of de novo depression-related consultations in people living with obesity.

## Method

### Data Source

The Clinical Practice Research Datalink (CPRD) was the source of data for this study. This is a national, UK-based, government-maintained research database, funded by the National Institute of Health Research and the Department of Health. It includes routinely collected primary care data representing around 10% of the national population. It has been validated for epidemiological research and is considered representative of the national population [11]. At the time of extraction, our dataset represented 674 practices up to 2016, with historical data available from 1950.

### Patient Inclusion Criteria

Patients over the age of 18, with a diagnosis of obesity based on a recorded BMI of greater than or equal to 30 kg/m^2^, were extracted from CPRD. These were restricted by year of first BMI between 1990 and 2016, age up to 70 and BMI less than 80. Cases were identified as patients who had undergone a bariatric procedure post 1990 based on Read codes (coded thesaurus of clinical terms to record findings and procedures in electronic medical records). Patients with their first BMI recorded after bariatric surgery were excluded.

### Case-Control Matching

Bariatric surgery patients (the cases) were matched 5:1 to patients who had not undergone surgery (controls) based on gender, first BMI (30–34.9, 35–39.9, 40–44.9, 45–49.9, 50 and over), age at first BMI (18–29.9, 30–34.9, 35–39.9, 40–44.9, 45–49.9, 50–54.9, 55–59.9, 60–64.9, 65–70) and year of first BMI (1990–1994, 1995–1999, 2000–2004, 2005–2009, 2010–2016). For cases, the index date was the bariatric surgery date. A control was assigned the index date of their matched case. From this population, cases and controls were identified as having pre-existing depression if there was a consultation recorded in which the patient had sought or received treatment for depression (herein described as *consultation related to depression*), defined as either at least a single Read code entry for depression (codes provided in supplementary data of this study) or prescription for antidepressant medication, in the 3 years before the index date.

The median time from first BMI to index date and from index date to end of CPRD coverage or death was compared between the surgical group and controls. The chi-square test was used to ensure matching had occurred for characteristics described above between the two groups, and to describe the differences in comorbidities before the index date identified by using the relevant Read codes—chronic renal failure, congestive heart failure, smoking, alcohol history, osteoarthritis, obstructive sleep apnea, hypertension and type II diabetes.

### Development of Depression After Bariatric Surgery

This analysis included all surgical cases and controls who did not have a consultation related to depression before the index date. Time to first consultation related to depression was determined where relevant. The remaining patients were censored at the end of follow-up, or date of death as provided by CPRD. Kaplan-Meier curves were constructed for surgical cases vs controls, with the log rank test used to demonstrate a significant difference in the cumulative risk of depression between the two groups. Hazard Cox regression modelling provided the risk of developing depression for the surgical group compared with controls, while including demographics and comorbidities with a statistical significant difference at *p* < 0.1. Landmark analyses were performed to reduce the effect of the surgical procedure with depression episodes, by considering depression Read codes or antidepressants only if present at least 1 year after the index date. Secondly, the impact of a change in weight (gain of + 5 kg, or a loss of more than 5 kg, compared with non-significant change (between + 5 and − 5 kg)) for those with at least 2-year follow-up was entered as an independent variable in the Cox regression analyses. Due to the low frequency of certain procedures, a sensitivity analysis based on the type of procedure was not possible.

### Resolution of Depression After Bariatric Surgery

This analysis included all surgical patients and controls who had a consultation relevant to depression before the index date. The time to the last consultation related to depression recorded in CPRD was determined for each patient or they were censored due to death or end of CPRD coverage. Kaplan-Meier analysis was used to describe the proportion of surgical patients and controls who had no further depression-related consultations at yearly intervals from the index date.

## Results

### Patient Cohort

**Fig. 1:**
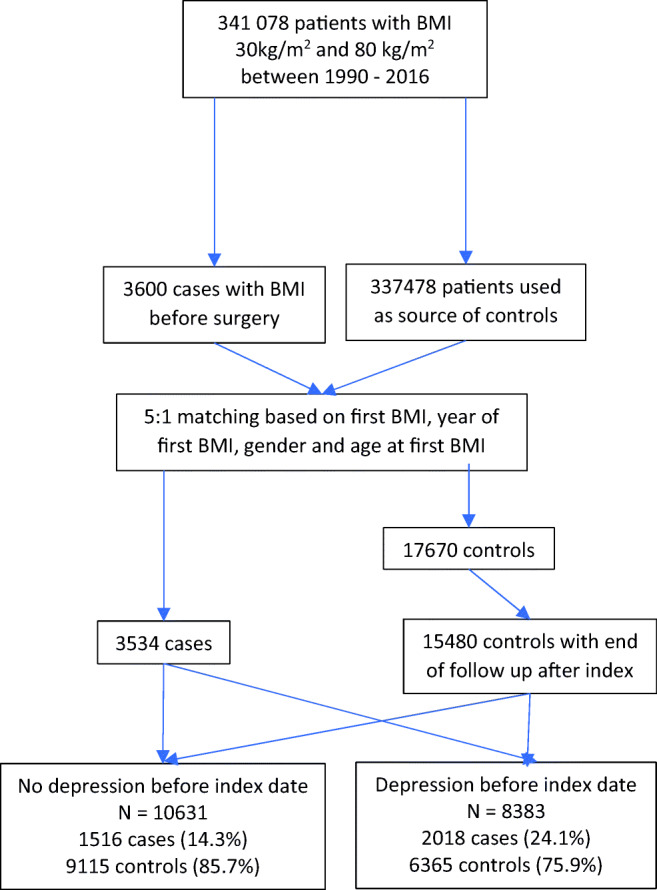
Flow chart to demonstrate case and control selection

In total, 363,037 patients over the age of 18, with a BMI of greater than or equal to 30 kg/m^2^, were extracted from CPRD. By applying the inclusion criteria described above, the dataset provided 341,078 patients. Of these, 3600 (1.1%) patients had undergone a bariatric procedure (see Fig. 1 for flow chart). A further 66 cases were excluded as they could not be matched successfully to five controls. After excluding patients whose assigned index date was after the end of CPRD coverage, 17,670 controls were restricted to 15,480. The median time from the first BMI to the index date was 8.4 years (IQR 4.3 to 13.9) for cases, compared with 8.9 years (IQR 4.8 to 14.2) for controls. From the index date to end of follow-up was a median of 4.2 years (IQR 2.2 to 6.3) for cases and 4.6 years (IQR 2.7 to 6.7) for controls.

A total of 8383 patients were deemed to have a consultation related to depression before the index date, of which 2018 (24.1%) had undergone surgery. The remaining 10,631 had no underlying depression before the index date, of which 1516 (14.3%) underwent bariatric surgery.

### Resolution of Depression After Bariatric Surgery

There was a statistically higher proportion of female patients in the control group (86.7%) compared with the surgical group (83.8%), but no difference in first BMI value, year of first BMI or age at first BMI (Table 1). The surgical group included a higher proportion of patients with hypertension, type II diabetes, osteoarthritis and obstructive sleep apnea (Table 1). There was no statistical difference in deaths between the two groups, with 35 (1.7%) deaths in the surgery group compared with 147 (2.3%) in the controls.

**Table 1:** Demographics, comorbidities and mortality for patients with a history of depression before the index date

		Bariatric surgery		*p*
		Cases	Controls	
Gender	Female	1692 (83.8)	5518 (86.7)	0.001
	Male	326 (16.2)	847 (13.3)	
Age at first BMI	18–29.9	602 (29.8)	1835 (28.8)	0.90
	30–34.9	374 (18.5)	1144 (18.0)	
	35–39.9	359 (17.8)	1171 (18.4)	
	40–44.9	275 (13.6)	967 (15.2)	
	45–49.9	210 (10.4)	681 (10.7)	
	50–54.9	122 (6.0)	381 (6.0)	
	55–59.9	45 (2.2)	123 (1.9)	
	60–64.9	26 (1.3)	51 (0.8)	
	65–70	5 (0.2)	12 (0.2)	
First BMI value	30–34.9	582 (28.8)	1850 (29.1)	0.72
	35–35.9	547 (27.1)	1645 (25.8)	
	40–44.9	436 (21.6)	1393 (21.9)	
	45–49.9	257 (12.7)	883 (13.9)	
	50 and over	196 (9.7)	594 (9.3)	
First BMI year	1990–1994	472 (23.4)	1432 (22.5)	0.30
	1995–1999	378 (18.7)	1150 (18.1)	
	2000–2004	618 (30.6)	1971 (31.0)	
	2005–2009	412 (20.4)	1406 (22.1)	
	2010 and later	138 (6.8)	406 (6.4)	
Comorbidities before index date	Cardiovascular	52 (2.6)	142 (2.2)	0.37
	CRF	47 (2.3)	157 (2.5)	0.73
	Hypertension	702 (34.8)	1672 (26.3)	< 0.0005
	Type II diabetes	500 (24.8)	1067 (16.8)	< 0.0005
	Osteoarthritis	356 (17.6)	896 (14.1)	< 0.0005
	OSA	308 (15.3)	230 (3.6)	< 0.0005
	Smoking history	729 (36.1)	2432 (38.2)	0.10
	Alcohol history	416 (20.6)	1214 (19.1)	0.13
Death recorded		35 (1.7)	147 (2.3)	0.12

Of the patients who underwent surgery, 52.4% had a further consultation related to depression compared with 39.8% of controls (*p* < 0.005).

**Fig. 2:**
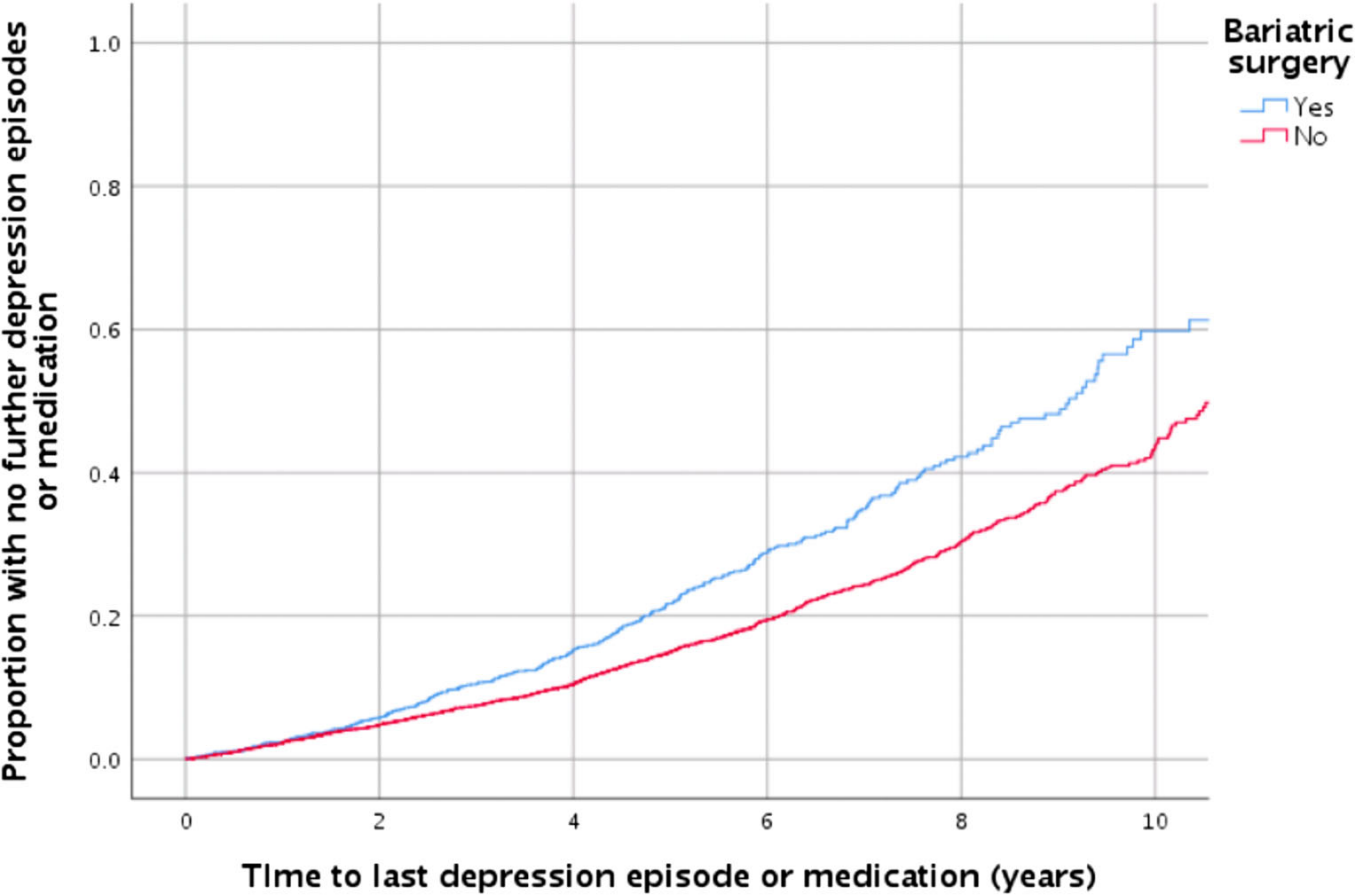
Kaplan-Meier analysis demonstrating the cumulative proportion of patients with no further consultations related to depression after the index date

As demonstrated by the Kaplan-Meier analysis (Fig. 2), a higher proportion of surgical patients had no further consultations related to depression (log rank chi-square 39.1, *p* < 0.005) at each yearly interval after the index date. By 5 years, just over 20% of surgical patients had no further consultations related to depression compared with 17% of controls.

### Development of Depression After Bariatric Surgery

There was no statistical difference in the first BMI, year of first BMI, gender or age at first BMI between the surgical and control groups (Table 2). A higher proportion of surgical patients had a diagnosis of hypertension, type II diabetes, osteoarthritis, obstructive sleep apnea, chronic renal failure, smoking history and heavy alcohol intake compared with controls (Table 2). There were 14 (0.9%) deaths in the surgical group compared with 149 (1.6%) of the controls (*p* = 0.04).

**Table 2:** Demographics, comorbidities and mortality for patients with no history of depression before the index date

		Bariatric surgery		*p*
		Cases	Controls	
Gender	Female	1119 (73.8)	6844 (75.1)	0.29
	Male	397 (26.2)	2271 (24.9)	
Age at first BMI	18–29.9	497 (32.8)	2943 (32.3)	0.73
	30–34.9	247 (16.3)	1592 (17.5)	
	35–39.9	254 (16.8)	1534 (16.8)	
	40–44.9	214 (14.1)	1175 (12.9)	
	45–49.9	161 (10.6)	936 (10.3)	
	50–54.9	90 (5.9)	554 (6.1)	
	55–59.9	35 (2.3)	224 (2.5)	
	60–64.9	16 (1.1)	137 (1.5)	
	65–70	2 (0.1)	20 (0.2)	
First BMI value	30–34.9	372 (24.5)	2342 (25.7)	0.35
	35–35.9	377 (24.9)	2394 (26.3)	
	40–44.9	365 (24.1)	2105 (23.1)	
	45–49.9	240 (15.8)	1300 (14.3)	
	50 and over	162 (10.7)	974 (10.7)	
First BMI year	1990–1994	261 (17.2)	1675 (18.4)	0.19
	1995–1999	253 (16.7)	1598 (17.5)	
	2000–2004	441 (29.1)	2685 (29.5)	
	2005–2009	443 (29.2)	2397 (26.3)	
	2010 and later	118 (7.8)	760 (8.3)	
Comorbidities before index date	Cardiovascular	20 (1.3)	123 (1.3)	0.93
	CRF	41 (2.7)	152 (1.7)	0.005
	Hypertension	465 (30.7)	2111 (23.2)	< 0.0005
	Type II diabetes	321 (21.2)	1193 (13.1)	< 0.0005
	Osteoarthritis	177 (11.7)	672 (7.4)	< 0.0005
	OSA	188 (12.4)	258 (2.8)	< 0.0005
	Smoking history	427 (28.2)	2286 (25.1)	0.01
	Alcohol history	212 (14.0)	974 (10.7)	< 0.0005
Death recorded		14 (0.9)	149 (1.6)	0.04

**Fig. 3:**
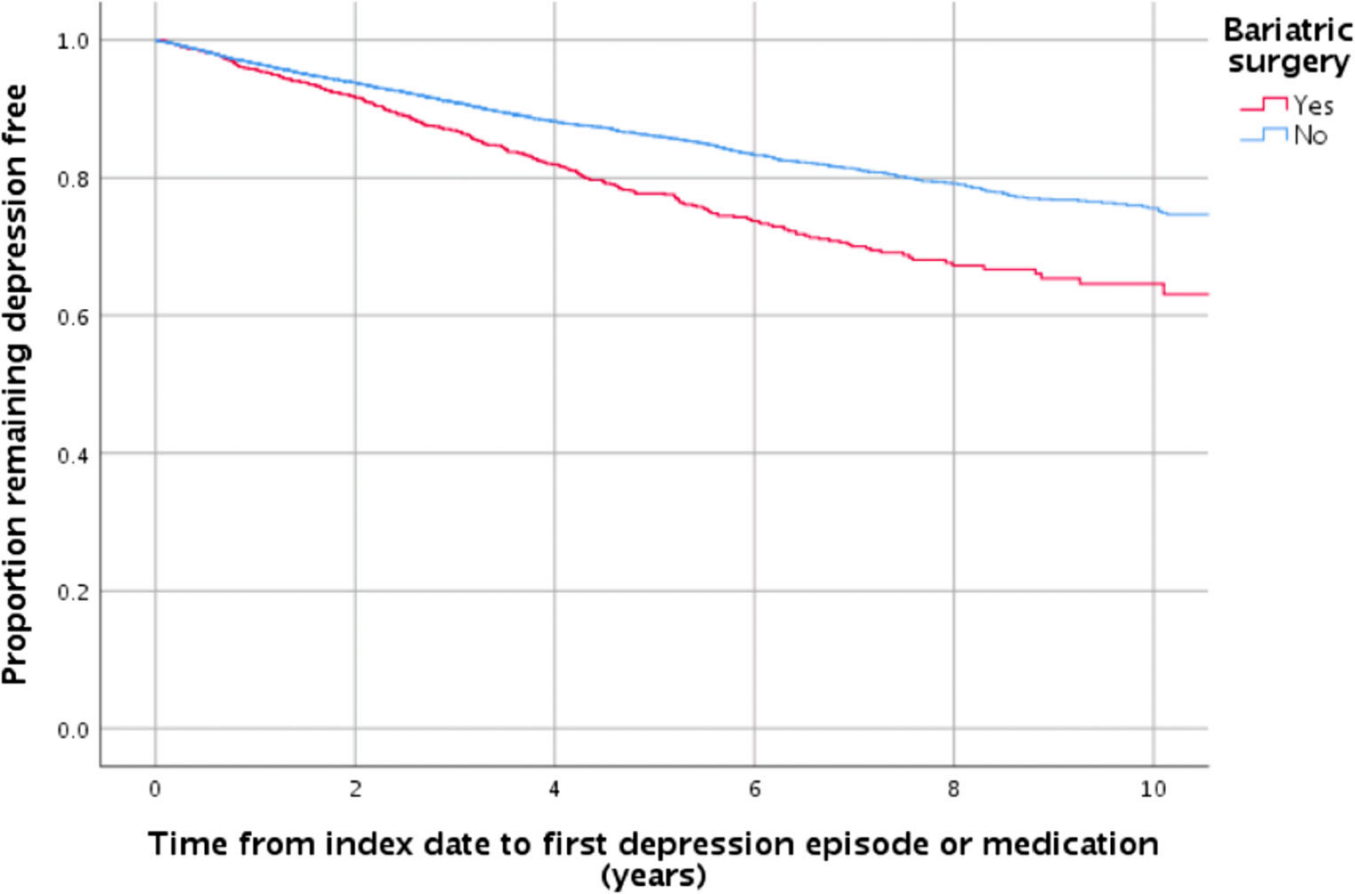
Kaplan-Meier analysis demonstrating the time to first consultation related to depression or medication for case (surgery) and controls (no surgery)

Of the surgical group, 308 (20.3%) had a consultation related to depression following surgery, compared with 1240 (13.6%) of controls (*p* < 0.005). As demonstrated by the Kaplan-Meier analysis, bariatric surgery was associated with an increased association of depression (Fig. 3) at all time points—at 5 years, 22% of surgical patients had a consultation related to depression compared with 15% the controls. After taking into consideration the comorbidities with a statistical difference at *p* < 0.1, surgery was associated with a 50% increased risk of developing depression (95% CI 1.32–1.71, *p* < 0.005).

By using a washout period of 12 months after the index date, the landmark analysis similarly showed an increased risk of depression de novo (HR of 1.51 (95% CI 1.33–1.72, *p* < 0.005)) for patients who underwent surgery. To investigate an association of weight loss with depression, 585 cases and 3343 controls were included as they had a weight recorded 2 to 4 years after the index date. Weight loss of more than 5% did not significantly alter the risk of developing depression (HR 1.11, 95% CI 0.81–1.53, *p* = 0.60). Although a weight gain of more than 5% tended towards an increased risk of depression (HR 1.22, 95% CI 0.98–1.50, *p* = 0.73), it failed to reach significance.

## Discussion

### Summary of Findings

Our study demonstrates bariatric surgery for patients with pre-existing depression is associated with a reduction in depression-related consultations. In contrast, surgery was associated with an increased risk of patients seeking medical attention for depression. Further analysis shows that the difference in depression diagnoses remained significant at 5 years post-index date between the surgical and control groups. This suggests that the changes shown are not an acute, reactive response to the burden of surgery, but a chronic change in patient wellbeing. Of note, our study design ensured the pre-index and follow-up period was similar between the surgical and control groups.

### Clinical Relevance

Due to the increased prevalence of depression in people living with obesity [12], it is fair to suggest that while some patients had no documented history of depression, undiagnosed or subclinical cases may have existed which came to light in the preoperative period. Involvement of bariatric psychologists preoperatively and follow-up care in the years following bariatric surgery would have aided the identification of new cases. Such a detection bias may explain the higher proportion of surgical patients who had depression in our study. Non-surgical patients are likely to have not had specialist support or assessment, including psychological, available in tier 3 obesity services [13]. An alternative explanation may be that people living with obesity who seek out surgery are distressed by their weight [14], or that the higher comorbidity burden in this group contributed to depression prevalence (although this was corrected for in the further analysis). In addition, patients who had a consultation related to depression or medication could have been experiencing a more severe level of depression than those who did not seek medical attention.

The increase in depression-related consultations after surgery in individuals without a pre-existing diagnosis of depression may also reflect the unique psycho-social challenges to which patients must acclimatise following bariatric surgery. The need for a period of ‘psychological adjustment’ post-surgery is well recognised [15] as changes in social eating behaviours, interpersonal relationships and physical appearance occur. While food is known to act as a potent mediator of the mesolimbic pathway in the brain, producing an enhanced mood when consumed and craved in its absence [16], the emotional significance of this cycle is heightened in individuals living with obesity [16]. Blunting of the dopaminergic activity within this system propagates excessive consumption of palatable food in order to replicate positive emotional responses of a similar magnitude [17, 18]. Changes in the anatomy of the gastrointestinal tract and subsequent dietary restrictions post-surgery mean previous excessive consumption of food is difficult. It stands that bariatric patients without a history of clinical depression may become symptomatic as food can no longer be used as a self-soothing or coping mechanism at times of increased stress or low mood. Instead, patients may experience a substitution of one compulsive behaviour for another, referred to as ‘addiction transfer’ [19] or ‘cross-addiction’ [20]. In these cases, patients turn to alterative (non-food) maladaptive coping mechanisms which further aggravate poor mental health, as evidenced by bariatric patients’ increased incidence of alcohol and substance abuse disorders [21] as well as self-harm [22, 23].

Within the scope of this study, we cannot comment on the level of psychological support given to patients’ post-surgery or the chronicity of symptoms but a recent qualitative study reported patients’ feeling of ‘abandonment’ due to insufficient multi-disciplinary support and variable availability of post-surgery support groups [24]. The complexity of the development of depression cannot simply be attributed to a change in weight, as our finding that this factor did not influence the likelihood of depression. This study highlights the role of the bariatric multi-disciplinary team in suggesting alternative coping strategies in the lead up to surgery and provide access to psychological support when necessary.

### Comparison to Other Studies

The conclusions in the literature about the benefit of bariatric surgery for depression are conflicting. Our finding that bariatric surgery leads to an earlier cessation in depressive symptoms is supported by multiple studies which show a decrease in depressive symptoms in a previously symptomatic population [25–27]. On the contrary, several studies have demonstrated an initial decrease in the rate of depression amongst patients with a positive history, followed by an increase in the following 3 years which may surpass pre-surgery levels [12, 27, 28]. One study which elicited a similar ‘fall-rebound’ pattern attempted to characterise patients’ changes in depression via additional questionnaires assessing mental and physical wellbeing and self-esteem [29]. Participants’ depression scores did not significantly change within the first 6 months post-bariatric surgery and the subsequent increase in depression scores was significantly and negatively related to self-esteem. The inverse was true for participants who reported decreased depression scores; a significant positive association was found with self-esteem scores. These authors concluded that increased depression post-surgery is related to novel factors detrimental to self-image—loose skin, social scrutiny, relationship breakdown [30]—rather than a relapse of pre-surgical causes of depression.

These factors may be behind the increase in de novo depression noted in this study, although it was not designed to investigate this. Also if obesity does lead to depression through common mediators such as inflammatory, psychosocial or poor chronic health, there is a possibility that depression may resolve after surgery. This may explain our finding that surgery was associated with an improvement for some with pre-existing depression (reviewer #2, comment #2).

### Strengths and Limitations

One of the crucial strengths of this study is the large study population; after exclusion criteria were met, the final cohort consisted of 3534 cases and 15,480 controls. Data was extracted from the UK CRPD, a database which was similarly utilised by Booth et al. to examine the impact of bariatric surgery on pre-existing clinical depression [12]. This study is a novel addition to the literature in this area as it is the only analysis to have included control-matched samples with and without pre-existing depression. Patients were also matched thoroughly, with a similar time from first BMI to index date and then from index date to termination of follow-up.

A limitation of this study is the inability to control certain aspects of patient care between case and control groups, leading to an element of confounding by indication. Patients being considered for surgery will tend to have more regular contact with healthcare professionals, and therefore depressed individuals are more likely to be recognised and diagnosed both before and after surgery. Additionally, diagnoses or episodes of depression may have been missed had they appeared exclusively in the free-text component which is not available to researchers. It is difficult to determine the severity and duration of depression as the Read coding relies on a subjective assessment of the patient’s mental health, with episodes of depression considered binary. Therefore, our data was not sensitive to distinctions between episodes of acute depression in previously asymptomatic individuals or exacerbations of chronic depression. Also, it is possible the medication identified as antidepressants may have been prescribed for non-depressive conditions such as chronic pain. It was not possible to account for other possible confounders such as level of deprivation, income or ethnicity as these variables were not available. Finally, this study identifies patients who sought intervention for their depression, which is likely to represent patients with worse severity of depression than those who do not see their primary care physician.

## Conclusion

Our study demonstrates the complexity of bariatric surgery as a management tool for depression in patients living with obesity. Under NICE Guidelines, medical indications for bariatric surgery include metabolic and cardiovascular comorbidities but do not consider the amelioration or prevention of psychological sequelae as part of its criteria. The findings of this study, supported by the bi-directional relationship between obesity and depression, encourage a considered approach by medical professionals in evaluating the enhanced benefits that bariatric surgery may provide to individuals with pre-existing depression.

## Supplementary Information

ESM 1 (DOCX 15 kb)
