# Supplementary material for: The Complex Association Between Bariatric Surgery and Depression: a National Nested-Control Study
Source: Obes Surg. 2021 Feb 3;31(5):1994–2001. doi: 10.1007/s11695-020-05201-z (PMC8041688; doi:10.1007/s11695-020-05201-z)
Supplement: Supplementary file 1 — (DOCX 15 kb) [file 11695_2020_5201_MOESM1_ESM.docx]

| Read code | Read term |
| --- | --- |
| Eu33200 | [X]Recurr depress disorder cur epi severe without psyc sympt |
| E113.00 | Recurrent major depressive episode |
| E112z00 | Single major depressive episode NOS |
| E11y.00 | Other and unspecified manic-depressive psychoses |
| Eu34114 | [X]Persistant anxiety depression |
| Eu34111 | [X]Depressive neurosis |
| Eu34112 | [X]Depressive personality disorder |
| Eu34113 | [X]Neurotic depression |
| 6659000 | Antidepressant drug treatment started |
| E112000 | Single major depressive episode, unspecified |
| Eu32000 | [X]Mild depressive episode |
| 1BT..00 | Depressed mood |
| E113400 | Recurrent major depressive episodes, severe, with psychosis |
| 1B17.00 | Depressed |
| Eu32z11 | [X]Depression NOS |
| Eu32.00 | [X]Depressive episode |
| E204.00 | Neurotic depression reactive type |
| Eu32100 | [X]Moderate depressive episode |
| Eu32z00 | [X]Depressive episode, unspecified |
| Eu32z14 | [X] Reactive depression NOS |
| E112.13 | Endogenous depression first episode |
| E112.14 | Endogenous depression |
| E2B1.00 | Chronic depression |
| E112.00 | Single major depressive episode |
| E135.00 | Agitated depression |
| E113700 | Recurrent depression |
| 9H91.00 | Depression medication review |
| 1285 | FH: Depression |
| Eu41200 | [X]Mixed anxiety and depressive disorder |
| Eu33.00 | [X]Recurrent depressive disorder |
| 1B1U.11 | Depressive symptoms |
| Eu32200 | [X]Severe depressive episode without psychotic symptoms |
| Eu32z12 | [X]Depressive disorder NOS |
| E112.12 | Endogenous depression first episode |
| Eu32400 | [X]Mild depression |
| Eu32.11 | [X]Single episode of depressive reaction |
| E113200 | Recurrent major depressive episodes, moderate |
| E112100 | Single major depressive episode, mild |
| Eu41211 | [X]Mild anxiety depression |
| E112300 | Single major depressive episode, severe, without psychosis |
| Eu32212 | [X]Single episode major depression w'out psychotic symptoms |
| Eu33000 | [X]Recurrent depressive disorder, current episode mild |
| E113100 | Recurrent major depressive episodes, mild |
| Eu33400 | [X]Recurrent depressive disorder, currently in remission |
| Eu32500 | [X]Major depression, mild |
| Eu32z13 | [X]Prolonged single episode of reactive depression |
| E113000 | Recurrent major depressive episodes, unspecified |
| Eu33212 | [X]Major depression, recurrent without psychotic symptoms |
| E112500 | Single major depressive episode, partial or unspec remission |
| E11y200 | Atypical depressive disorder |
| Eu33y00 | [X]Other recurrent depressive disorders |
